# Fear of progression, loneliness, and hope in patients with gastrointestinal cancer: a cross-sectional relational study

**DOI:** 10.3389/fpsyg.2023.1279561

**Published:** 2024-01-05

**Authors:** Yanjun Li, Tian Xiao, Haiyan Liao, Haimei Qu, Pan Ren, Xiaoju Chen

**Affiliations:** ^1^School of Nursing, Chengdu Medical College, Chengdu, Sichuan, China; ^2^Department of Nursing, Suining Central Hospital, Suining, Sichuan, China; ^3^Department of Oncology, Chengdu Pidu District People’s Hospital, Chengdu, Sichuan, China

**Keywords:** gastrointestinal neoplasms, fear of progression, hope, loneliness, cancer nursing

## Abstract

**Introduction:**

In recent years, fear of disease progression (FoP) has become one of the most common psychological problems in cancer patients. However, there are fewer studies on the FoP in patients with gastrointestinal tumors. We aimed to assess the level of FoP in patients with gastrointestinal tumors and analyze the factors related to FoP. We also aimed to examine the relationship among loneliness, hope and FoP in patients with gastrointestinal cancer.

**Methods:**

A cross-sectional survey was conducted on three Grade A hospitals in southwestern China from November 2021 to July 2022. The demographic and clinical characteristics questionnaire, Fear of Disease Progression Scale (FoP-Q-SF), Cancer Loneliness Scale (CLS), and Herth Hope Index (HHI) were included in this study. Data analysis included descriptive statistics, independent samples *t*-tests, one-way analysis of variance, and multiple linear regression analysis.

**Results:**

In total, 245 gastrointestinal cancer patients participated in this study. The average (standard deviation) FoP score in patients was 32.94 ± 10.64. In total, 245 gastrointestinal cancer patients participated in this study. The average (standard deviation) FoP score in patients was 32.94  ±  10.64. The average score of CLS was 17.65  ±  6.71, and that for the HHI was 31.27  ±  7.73. Pearson correlation analysis showed that FoP was negatively significant correlated with hope level (*r* = −0.522) and FoP was positively significant correlated with loneliness (*r* = 0.545). Linear regression analysis showed that educational level, age, living condition, hope, and loneliness were the significant predictors of FoP and explained 53.10% of the variability in FoP (*F* = 16.372).

**Conclusion:**

Findings highlight the need to strengthen attention to FoP in gastrointestinal cancer patients. Our study showed that gastrointestinal cancer patients who have a high school education, are age 45 to 59, live alone, high level of loneliness, and low level of hope have higher FoP. Medical staff should enhance clinical screening of FoP and consider the formulation of relevant interventions for high-risk groups to reduce loneliness among patients, raise their hope level, and reduce their FoP.

## Introduction

According to the 2020 Global Cancer Statistical Report, the number of new cases and deaths owing gastrointestinal neoplasms ranks among the highest worldwide (Sung et al., 2021). Gastrointestinal neoplasms have the characteristics of a short course, rapid development, early metastasis, and generally poor prognosis; thus, these cancers have become a serious global public health problem (Hamashima, 2014). According to different stages of gastrointestinal neoplasms, a series of treatments are available, such as surgery, radiotherapy, and chemotherapy, but all these have adverse effects on patients (Numico et al., 2015). Due to the special physiological characteristics of patients with gastrointestinal tumors, they are more likely to have adverse reactions such as nausea, vomiting, renal insufficiency, and radiation enteritis during cancer treatment (van den Boorn et al., 2020). Moreover, the long-term sequelae associated with cancer treatments can affect the social life of patients with gastrointestinal tumors, resulting in substantial psychosocial pressure and distress (Kim and Yoo, 2021). Research has shown that fear of progression (FoP) is one of the most frequent distress symptoms in people with cancer (Herschbach and Dinkel, 2014).

FoP has been proposed by Dankert et al. (2003), as referring to an individual’s fear of everything related to his or her real-life disease, specifically defined as the various biopsychosocial consequences of the fear of disease progression or the fear of disease recurrence. It is reported that 33 to 96% of cancer patients worldwide have FoP, and the proportion with severe fear reaches 87% (Lebel et al., 2016). FoP has become one of the most common psychological problems in patients with cancer, and FoP is now included in the international definition of fear of cancer recurrence (FCR; Youssef et al., 2021). FCR is fear or apprehension in cancer survivors about the possibility of cancer recurrence, progression, or metastasis at the primary site or elsewhere (Barrows et al., 2020). It is clear that the relevant defining features of the two concepts are comparable and identical in nature Herschbach and Dinkel (2014). In the present paper, we treat FoP and FCR as equivalent from the perspective of tumor psychology.

Many scholars have interpreted the influencing factors of FoP from different perspectives. Some studies have shown that FoP in cancer patients is related to sex or gender (Pang and Humphris, 2021), age (Lim and Humphris, 2020), educational level (Thewes et al., 2018), and marital status (Thewes et al., 2016). In a cross-sectional study, high levels of FoP in Korean survivors of gastric cancer were associated with poor social support, functional decline, fatigue, pain, sleep problems, anxiety, and depression, regardless of sex, type of cancer treatment, duration, or disease stage (Shin et al., 2022). Previous studies have found that colorectal cancer patients with high levels of FoP may face higher levels of psychological distress, post-traumatic stress symptoms, and lower quality of life (Custers et al., 2016). FoP is therefore affected by many factors and can seriously affect the physical and mental health of patients with cancer. Based on this, we aimed to explore multiple perspectives to determine the influence of various factors on FoP in patients with gastrointestinal neoplasms in China.

Loneliness is another important negative emotion that is common in cancer patients. As a common chronic stressor, loneliness accelerates the progression of cancer (Dai et al., 2020). Loneliness refers to the emotional experience of social isolation due to a subjective desire for social interactions that are higher than the actual level of socialization (Ross et al., 2020). Loneliness in cancer patients is related to the specific cancer experience of patients. Studies have shown that patients with cancer have a greater risk of loneliness (Gray et al., 2020). Research on loneliness in cancer patients has been conducted for many years. A 5-year follow-up study confirmed that high levels of loneliness can cause patients great distress (Drageset et al., 2013). Cancer patients with high levels of loneliness also have more severe depressive symptoms, often accompanied by higher mortality. A cross-sectional descriptive correlation study found that adult patients with cancer felt moderate levels of loneliness (Kavalal and Koc, 2021). A population-based national study showed that the average age at diagnosis of digestive tract tumors in China is 55.20 years, and most patients were adults (Xu et al., 2021). Meanwhile, it has been shown that there is an association between loneliness and FoP in breast cancer patients, with patients with high levels of loneliness having a higher risk of psychological problems (Li et al., 2022). Therefore, the relationship between the level of loneliness and FoP in patients with gastrointestinal tumors deserves further exploration. In this study, we investigated the correlation between loneliness and FoP among patients with gastrointestinal tumors in China.

Hope plays a significant role in the field of positive psychology. Hope can help cancer patients better cope with the disease, promote healthy behaviors, and reduce the occurrence of depression (Corn et al., 2020). Previous research demonstrated that maintaining a positive and optimistic mood helps alleviate patients’ fear of disease progression (Otto et al., 2016). Research indicated that even patients with advanced cancer can possess substantial levels of hope, predominantly related to a potential cure and expectations regarding quality of life (Daneault et al., 2016). An observational cross-sectional study showed that when cancer patients have high levels of hope, they have a positive attitude toward treatment and a high degree of subjective cooperation (Ozen et al., 2020). Additionally, a study of long-term survivors of colorectal cancer showed that patients with enterostomy experienced different levels of psycho-emotional conditions, decreased levels of hope, and increased levels of anxiety and depression (Kunitake et al., 2017).

The Herth’s theory of hope holds that the essence of hope is belief, centered on positive goals, which gives patients sustained motivation. As a common psychological state in cancer patients coping with the disease, FoP can also affect levels of hope (Starreveld et al., 2018). Loneliness is thought to be an important psychosocial factor associated with cancer severity (Dai et al., 2020). Therefore, the research questions addressed were: (a) What is the FoP level in patients with gastrointestinal tumors? (b) How does the level of FoP vary according to demographic and disease-related variables? (c) What is the relationship between FoP, loneliness, and hope?

## Materials and methods

### Study setting and participants

A cross-sectional relational design was used in this study. The study was conducted between November 2021 and July 2022 in the oncology departments of three Grade A hospitals in southwest China. We guaranteed patient confidentiality and the right to withdraw from the study at any time. The STROBE guidelines for cross-sectional studies were adhered to in this study (Supplementary Table S1). Study participants were recruited from the oncology departments of three Grade A hospitals in southwest China using a convenience sampling method. The inclusion criteria were as follows: (a) diagnosed with gastrointestinal tumors in clinicopathological imaging and hospitalized for cancer treatment; (b) aged 18–80 years; and (c) provided informed consent. The exclusion criteria were as follows: (a) other types of cancer; (b) lacking the ability to communicate effectively with others; or (c) current participation in other studies. G*Power Version 3.1 for multiple linear regression was utilized to calculate the sample size (Faul et al., 2007). Based on a medium effect size of 0.15, alpha of 0.05, power of 0.95 and 23 predictors, the sample size was determined 234. The sample of 245 met the criteria of this study.

### Ethical consideration

An informed consent was signed and that the study was approved by the hospital’s ethics committee (approval number: 2021CYFYIRB-BA-60-01).

### Data collection

Study recruitment information was posted by the research staff at the time of admission. The investigators screened inpatients with digestive system cancer who met the eligibility requirements in person on the hospital ward. Before the formal survey, we fully explained the purpose and content of the study to patients again and then distributed questionnaires. The data collection method was a combination of electronic medical record review and a paper questionnaire survey. Approximately 20 min were required to complete the questionnaire. After completing the questionnaire, the investigators checked the quality of the questionnaire promptly, asked patients about missing or unclear items, and corrected and completed the information to ensure the quality of the questionnaire. We made sure to avoid any inductive language during questioning. Research team members then extracted demographic and disease-related data from patients’ electronic medical records. A total of 250 questionnaires were distributed. Five patients had to stop the survey because of physical discomfort. Ultimately, 245 completed questionnaires were collected.

### Measures

#### Demographic and disease-related characteristics

In the present study, we collected sociodemographic data using 12 items, including sex, age, educational level, marital status, geographic area, living condition (living alone or with family members), the number of escorts, insurance type, occupational status, income (RMB/month), family history of cancer, and religious beliefs. Four items were used to investigate disease-related features, including tumor stage, time of diagnosis (month), disease type, and type of treatment.

#### Fear of Progression Questionnaire-Short Form

FoP was measured using the Chinese version of the Fear of Progression Questionnaire-Short Form (FoP-Q-SF). This short-form questionnaire was translated into Chinese by Wu et al. (2015). The short-form questionnaire includes a total of 12 items in two dimensions, the physiological dimension (6 items) and social dimension (6 items), based on a 5-point Likert scale (1 = strongly disagree, 5 = strongly agree), for a total score ranging from 12 to 60. Higher scores indicate higher levels of FoP. A score of 20–31 indicates mild fear, 32–38 moderate fear, and 39–60 indicates severe fear (Zhang et al., 2022a). The total Cronbach’s α for the Chinese version of the FoP-Q-SF was 0.883. In the current study, the Cronbach’ s α was 0.905 in total for the short-form questionnaire.

#### Cancer Loneliness Scale

The Chinese version of the Cancer Loneliness Scale (CLS) was translated by Cui and Sun (2020). The scale is used to evaluate loneliness among Chinese patients with gastrointestinal neoplasms. This scale has seven items across a single dimension graded on a 6-point Likert scale (1 = strongly disagree, 6 = strongly agree), for a total score of 7–35. A higher score on the CLS indicates more serious loneliness. The Cronbach’s α coefficient of the Chinese version of CLS was 0.912 (Cui and Sun, 2020). In the current study, the Cronbach’s α was 0.881, indicating good reliability and validity.

#### Herth Hope Index

Hope was measured using the Chinese version of the Herth Hope Index (HHI) by Zhao and Wang (2000). This instrument includes 12 items in three dimensions: temporality and future, positive readiness and expectancy, and interconnectedness. Each item on the HHI is scored on a scale from 1 to 4, with 1 being “strongly disagree” and 4 being “strongly agree,” for a total score of 12–48. A score of 12–23 indicates a low level of hope, 24–35 a moderate level of hope, and 36–48 indicates a high level of hope. The total Cronbach’s α for the Chinese version of the HHI was 0.85 (Zhao and Wang, 2000). In the current study, the Cronbach’s α for the overall HHI was 0.910.

### Data analysis

All analyses were performed using IBM SPSS version 26 (IBM Corp., Armonk, NY, United States). Descriptive statistics are presented as frequencies and scores. The *t*-test and analysis of variance (ANOVA) were used to test differences in FoP between patients with different demographic and clinical variables. Pearson’s correlation was used to analyze the correlation among three variables: FoP, loneliness, and hope. At the same time, we set up virtual coding (Supplementary Table S2), and all dummy variables were entered into the regression equation using the input method; the other variables were entered into the regression equation using the stepwise method. Multiple linear regression analysis was used to identify important predictors of FoP among patients with gastrointestinal tumors. *p* < 0.05 was considered statistically significant.

## Results

### Descriptive analysis

The main characteristics of participants can be seen in Table 1. A total of 245 patients were recruited for this study. Most participants (*n* = 149, 60.8%) were men. In addition, 127 participants (51.80%) were aged 60–80 years, and 105 (42.90%) were aged 45–59 years. Furthermore, most participants (*n* = 212, 86.50%) were married; 109 patients (44.50%) lived in a rural area and 136 (55.50%) lived in an urban area. Regarding disease-related characteristics, most patients (*n* = 75, 30.60%) had been diagnosed between 9 and 12 months earlier. In addition, 39.2% of patients (*n* = 96) had stage IV tumors, and most patients (*n* = 57, 22.90%) were treated with concurrent chemoradiotherapy. The most common cancer (*n* = 80, 32.70%) was colorectal cancer, followed by gastric (*n* = 56, 22.90%), esophageal (*n* = 47, 19.20%), and liver cancer (*n* = 33, 13.50%).

**Table 1:** FoP and associations with demographic and clinical variables characteristics (*N* = 245).

Variables	Group	Total *n* (%)	Scores	F/t	*p*
*Gender*					
	Male	149 (60.80)	32.50 ± 11.03	t = −0.810	0.419
	Female	96 (39.20)	33.63 ± 10.03		
*Age (years)*					
	<45	13 (5.30)	36.69 ± 11.61	*F* = 8.150	0.000
	45 ~ 59	105 (42.90)	35.57 ± 10.78		
	60 ~ 80	127 (51.80)	30.38 ± 9.84		
*Educational level*					
	Primary or below	124 (50.60)	32.58 ± 10.14	*F* = 4.422	0.005
	Junior high school	70 (28.60)	33.94 ± 10.88		
	High school	26 (10.60)	37.46 ± 11.59		
	College or above	25 (10.20)	27.20 ± 9.19		
*Marital status*					
	Married	212 (86.50)	31.76 ± 9.39	*F* = 23.161	0.000
	Widowhood/Widowerhood	24 (9.80)	35.46 ± 13.76		
	Divorce	9 (3.70)	54.00 ± 5.57		
*Pain level*					
	Painless	138 (56.30)	30.99 ± 9.70	*F* = 6.879	0.000
	Mild pain	91 (37.10)	34.33 ± 10.68		
	Moderate pain	12 (4.90)	39.67 ± 13.08		
	Severe pain	4 (1.60)	48.25 ± 11.76		
*Geographic area*					
	Rural	109 (44.50)	34.94 ± 11.31	*t* = 2.662	0.008
	Urban	136 (55.50)	31.34 ± 9.82		
*Living status*					
	Live alone	19 (7.80)	52.95 ± 7.66	*t* = −10.167	0.000
	Live with family	226 (92.20)	31.26 ± 9.03		
*Number of escorts*					
	0	72 (29.40)	32.25 ± 12.87	*t* = 1.950	0.054
	1 ~ 2	173 (70.60)	31.98 ± 9.44		
*Insurance type*					
	Social insurance	150 (61.20)	31.85 ± 10.54	*t* = −2.019	0.045
	Rural cooperative medical insurance	95 (38.80)	34.65 ± 10.63		
*Occupational status*					
	Farmer	79 (32.20)	32.73 ± 11.05	*F* = 3.547	0.015
	On the job	32 (13.10)	33.84 ± 10.68		
	Unemployment	63 (25.70)	35.94 ± 11.23		
	Retirement	71 (29.00)	30.10 ± 8.93		
*Income (RMB/month)*					
	<1,000	73 (29.80)	33.10 ± 9.78	*F* = 6.159	0.002
	1,000 ~ 2,999	109 (44.50)	34.99 ± 11.09		
	≥3,000	63 (25.70)	29.21 ± 9.95		
*Tumor stage*					
	II	69 (28.20)	33.35 ± 11.15	*F* = 1.161	0.315
	III	80 (32.70)	34.08 ± 11.30		
	IV	96 (39.20)	31.70 ± 9.64		
*Time of diagnosis (month)*					
	1 ~ 4	43 (17.60)	31.21 ± 11.39	*F* = 5.36	0.658
	5 ~ 8	57 (23.30)	33.79 ± 11.45		
	9 ~ 12	75 (30.60)	32.88 ± 10.12		
	>12	70 (28.60)	33.37 ± 10.13		
*Family history of cancer*					
	Yes	61 (24.90)	34.56 ± 10.09	*t* = 1.373	0.171
	No	184 (75.10)	32.40 ± 10.79		
*Religious belief*					
	Buddhism	16 (6.60)	29.06 ± 7.46	*F* = 1.601	0.204
	Christianity	2 (0.80)	40.50 ± 19.09		
	No	226 (92.60)	33.12 ± 10.75		
*Type of disease*					
	Esophageal cancer	47 (19.20)	32.87 ± 9.49	*F* = 0.956	0.456
	Rectal cancer	47 (19.20)	34.02 ± 9.83		
	Colon cancer	33 (13.50)	35.21 ± 13.00		
	Liver cancer	33 (13.50)	33.00 ± 12.29		
	Gastric cancer	56 (22.90)	30.50 ± 10.45		
	Pancreatic cancer	22 (9.00)	34.32 ± 9.69		
	Cholangiocarcinoma	7 (2.90)	30.29 ± 5.41		
*Treatment*					
	Chemotherapy + radiotherapy	57 (23.30)	35.35 ± 11.25	*F* = 1.547	0.163
	Surgery + chemotherapy/radiotherapy	47 (19.20)	33.83 ± 9.59		
	Surgery + chemotherapy	56 (22.90)	31.68 ± 10.96		
	Surgery + radiotherapy	7 (2.90)	30.29 ± 5.06		
	Only surgery	8 (3.30)	29.25 ± 6.80		
	Only chemotherapy	55 (22.40)	30.87 ± 10.50		
	Only radiotherapy	15 (6.10)	36.47 ± 12.64		

### Univariate analysis

FoP varied significantly according to age, education level, marital status, pain score, geographic area, living condition, type of insurance, occupational status, and average monthly income. However, there were no significant differences for FoP among participants by sex, tumor stage, tumor type, and family history of cancer (Table 1).

### Correlation analysis

As presented in Table 2, the average score for FoP was 32.94 ± 10.64, which indicated a moderate level of FoP. The average score for the CLS was 17.65 ± 6.71, and HHI showed an average score of 31.27 ± 7.73. Furthermore, Table 3 showed that FoP was negatively significant correlated with the level of hope (*r* = −0.522, *p* < 0.01) and positively significant correlated with loneliness (*r* = 0.545, *p* < 0.01). Hope was inversely correlated with loneliness (*r* = −0.625, *p* < 0.01). This further suggested that the higher the patient’s level of hope, the lower their level of FoP, as well as the higher the level of loneliness, the higher the patient’s degree of FoP.

**Table 2:** Scores of the Fear of Progression Questionnaire-Short Form, Cancer Loneliness Scale, Herth Hope Index (*N* = 245).

Variables (items no.)	Mean ± SD	Min	Max	Skewness	Kurtosis
FoP-Q-SF	32.94 ± 10.64	18	60	0.985	0.162
Cancer Loneliness Scale	17.65 ± 6.71	13	48	0.290	−0.644
Herth Hope Index	31.27 ± 7.73	7	33	−0.158	−0.690

SD, standard deviation; FoP-Q-SF, Fear of Progression Questionnaire Short Form.

**Table 3:** Pearson’s correlation among FoP, loneliness, and hope (*N* = 245).

	FoP-Q-SF	Physiological dimension	Social dimension	T	P	I	HHI	CLS
FoP-Q-SF	1							
Physiological dimension	0.910^**^	1						
Social dimension	0.937^**^	0.708^**^	1					
T	−0.479^**^	−0.509^**^	−0.387^**^	1				
P	−0.389^**^	−0.361^**^	−0.358^**^	0.680^**^	1			
I	−0.540^**^	−0.541^**^	−0.465^**^	0.742^**^	0.719^**^	1		
HHI	−0.522^**^	−0.522^**^	−0.450^**^	0.885^**^	0.893^**^	0.919^**^	1	
CLS	0.545^**^	0.533^**^	0.479^**^	−0.584^**^	−0.505^**^	−0.601^**^	−0.625^**^	1

^**^p < 0.01; FoP-Q-SF, Fear of Progression Questionnaire Short Form; HHI, Herth Hope Index; CLS, Cancer Loneliness Scale; T, temporality and future; P, positive readiness and expectancy; I, interconnectedness.

### Regression analysis

Based on the ANOVA, *t*-test, and Pearson correlation analysis results, we entered the significant variables into the regression equation model. As shown in Table 4, education level, age, living status, level of hope, and loneliness were important predictors of FoP in patients with gastrointestinal tumors. Among them, five variables, high school education level, 45–59 years old, living alone, low level of hope, and high level of loneliness, were statistically significant in the regression model, explaining 53.10% of the variance in FoP (*F* = 16.372).

**Table 4:** Multiple regression results with FoP as dependent variables (*N* = 245).

Variables	*β*	SE	*β*′	*t*	*p*	Beta (CI 95%)
Constant	36.542	4.152		8.801	0.000	28.359 ~ 44.722
Educational level						
Junior high school	0.322	1.314	0.014	0.245	0.807	−2.268 ~ 2.912
High school	4.119	1.789	0.119	2.302	0.022	0.594 ~ 7.645
College or above	−0.262	2.258	−0.007	−0.116	0.908	−4.712 ~ 4.188
Age (years)						
45 ~ 59	3.767	1.250	0.176	3.014	0.003	1.304 ~ 6.229
<45	3.533	2.715	0.075	1.301	0.195	−1.817 ~ 8.884
Marital status						
Widowhood/Widowerhood	−2.842	1.823	−0.080	−1.559	0.120	−6.434 ~ 0.750
Divorce	−0.635	3.198	−0.011	−0.199	0.843	−6.938 ~ 5.667
NRS scores						
Mild pain	1.796	1.050	0.082	1.709	0.089	−0.274 ~ 3.865
Moderate pain	4.184	2.241	0.085	1.867	0.063	−0.232 ~ 8.601
Severe pain	−5.797	4.193	−0.069	−1.383	0.168	−14.060 ~ 2.466
Income (RMB/month)						
1,000 ~ 2,999	0.418	1.236	0.020	0.338	0.736	−2.018 ~ 2.854
≥3,000	−0.258	1.773	−0.011	−0.145	0.885	−3.752 ~ 3.236
Occupational status						
On the job	2.701	2.001	0.086	1.350	0.178	−1.242 ~ 6.645
Farmer	0.861	1.435	0.038	0.600	0.549	−1.967 ~ 3.688
Retirement	−0.017	1.530	−0.001	−0.011	0.991	−3.032 ~ 2.999
Living status	17.853	2.432	0.450	7.341	0.000	13.061 ~ 22.646
HHI score	−0.422	0.087	−0.307	−4.835	0.000	−0.594 ~ −0.250
CLS score	0.266	0.098	0.168	2.729	0.007	0.074 ~ 0.458

*R*^2^ = 0.566, adjusted *R*^2^ = 0.531, *F* = 16.372, *p* < 0.001. β, standardized estimate; SE, standard error; β′, non-standardized estimate CI 95% = confidence interval 95%; FoP-Q-SF, Fear of Progression Questionnaire Short Form; HHI, Herth Hope Index; CLS, Cancer Loneliness Scale; NRS, Numerical Rating Scale.

## Discussion

In this study, we measured the levels of FoP in patients with gastrointestinal tumors, analyzed the influencing factors of FOP, and explored the relationship between hope, loneliness, and FoP. The findings confirmed that patients with gastrointestinal tumors had moderate levels of FoP, that hope levels and loneliness were significantly associated with FoP, and that patients’ FoP levels were also influenced by a variety of other factors.

### Current status of FoP among gastrointestinal cancer patients

In our study, the average score (standard deviation) for FoP among patients with gastrointestinal tumors was 32.94 ± 10.64. However, it is worth mentioning that a previous study reported 335 patients with mixed cancer diagnosis with a lower FOP score (16.70 ± 0.46) than our study (Sarkar et al., 2015). The reason for this may be that 71.50% of patients had received a cancer diagnosis less than 1 year earlier in our study, and newly diagnosed patients had greater fear and worry about the disease. In addition, patients with gastrointestinal tumors have a high symptom burden, which also increases patients’ FoP. We also found that on the physical dimension, the patients’ greatest fear was that the treatment and medication would damage my body (mean score, 3.65), which was similar to the findings of other studies (Hong et al., 2020). Patients with gastrointestinal tumors mainly rely on surgical treatment, chemotherapy, and radiation therapy. Postoperative complications and the toxic side effects of radiotherapy and chemotherapy can exacerbate patients’ fear of disease treatment and medication. Therefore, although the effects of time to cancer diagnosis and treatment modality on FoP were not statistically significant in the univariate analysis of this study, we should still pay attention to the effects of these clinically relevant variables on FoP in patients with gastrointestinal tumors. Moreover, clinical staff should provide tailored psychosocial care to patients with gastrointestinal cancers, especially close to the time of diagnosis. For example, patients should be educated about disease treatment and preventive measures for the adverse effects of radiotherapy to reduce their fear level and psychological burden.

### Influencing factors of FoP for gastrointestinal cancer patients

Family is an important factor influencing FoP in patients with gastrointestinal tumors. We found that in the social dimension, the item with the highest score was worrying about what would happen to the patient’s family (average score, 3.40). In a survey of Chinese patients with lung cancer (Kuang et al., 2022), the highest score was also for worry about the family, with an average score of 3.31, which is similar to our research results. A possible explanation for this phenomenon is that FoP is influenced by the concept of family in the traditional culture of Confucianism in China. Today, there is still a strong family concept in Chinese society, and families are closely linked. There are often two or three generations living together in the same household (Yiu et al., 2021). On the one hand, patients with cancer worry that treatment of the disease will increase the financial burden on family members; on the other hand, patients worry that disease progression or death will bring irreparable psychological trauma to their family members (Kang et al., 2022). Therefore, this item has the highest score. Interestingly, a study of leukemia patients in Germany (Thiele et al., 2020) and another study of cervical cancer patients in Thailand (Hanprasertpong et al., 2017). Also showed that “worrying about my family” was the highest scoring entry on the FoP. This illustrates that in Asian as well as European countries, the family is of great concern to cancer patients, who may be as worried about their family as they are about themselves. As an important ethical standard in traditional Chinese culture, filial piety is considered the most important cultural foundation for regulating intergenerational relationships in Chinese families. Through the process and function of socialization, filial piety is internalized in people’s conscious awareness, guiding and constraining obedience and respect toward parents and elders (Bedford and Yeh, 2019). In addition, in terms of the residential situation, patients who lived alone had higher FoP levels than those who lived with family members. Patients who live alone usually receive less social support from family and friends and have a greater FoP (Zhong et al., 2022). Patients living alone must also bear the physical and psychological pain caused by cancer alone, so they pay greater attention to progression or recurrence of the disease. On the contrary, patients who live with their family members can communicate with family when they encounter problems owing to disease progression, which will help reduce the patient’s psychological pain and pressure and reduce their fear levels (Yeung and Lu, 2022). Therefore, medical staff should encourage patients to communicate with their family and friends to improve loneliness, help patients obtain more sources of social support, and help them deal with negative emotions.

Age is an influencing factor in FoP among patients with gastrointestinal tumors. In this study, the FoP score of patients aged less than 45 years (36.69 ± 11.61), 45–59 years (35.57 ± 10.78), and 60–80 years (30.38 ± 9.84) gradually decreased with age. The FoP scores of young and middle-aged patients in our study were lower than the FoP scores in a survey among adult cancer patients in Germany (Richter et al., 2022). The possible reason for this could be differences in the age group divisions of participants in the different studies. Richter limited the age range of the survey participants to 15–39 years old; in the current study, we considered young cancer patients to be those less than 45 years old. Our study results showed that compared with patients aged 45–59 years, those aged 60–80 years had less FoP. This may be related to coping strategies adopted by patients of different ages (Hernandez et al., 2019). The older the patient and the richer their social experience, the better at regulating their emotions and the more actively and calmly they can face a disease diagnosis, leading to lower FoP scores (Richter et al., 2022). However, the reason for the higher levels of FoP in young and middle-aged patients may be related to earlier incidence of gastrointestinal tumors (Arnold et al., 2020). Alternatively, these results may also be because young and middle-aged patients take on more professional and family responsibilities. After a cancer diagnosis, these responsibilities do not diminish, leading to great psychological pressure as people may lose their job due to cancer treatment, which can increase their financial burden (Maheu et al., 2021). This can ultimately lead to higher FoP levels in this population. Therefore, clinical medical staff should pay greater attention to the economic situation of middle-aged and young patients with gastrointestinal cancer and guide them in easily accessing medical resources. For example, hospitals can work with relevant departments to provide financial assistance (such as reducing prescription drug costs or non-medical daily expenses) and conduct educational lectures (such as improving financial literacy, debt planning, and registering and optimizing health insurance; Smith et al., 2022).

This study also found that compared to patients who had elementary education levels, patients with high school education levels had a higher FoP level in relation to gastrointestinal cancer. This is consistent with the conclusion of a study conducted by Mahendran et al. (2021) and may be because patients with high literacy levels actively seek information about a disease, treatment, and prognosis, thereby increasing their awareness about cancer recurrence and risk factors; this, in turn, can lead to more catastrophic thinking and increase the patient’s level of fear.

### The relationship between loneliness and FoP

Loneliness in patients with gastrointestinal tumors is positively significant correlated with FoP, that is, the higher the level of loneliness, the higher the patient’s degree of FoP. This finding aligns with the conclusions of a study conducted among patients diagnosed with head and neck cancer and their caregivers (Maguire et al., 2017). In both the Western and Chinese cultural contexts, lonely patients develop emotional expression disorders when they are unable to freely express their emotions owing to a lack of social contact with others, leading to increased levels of phobia disorders (Barracliffe et al., 2018). The cognitive theory of loneliness states that loneliness can harm an individual’s cognition and behavior (Cacioppo and Hawkley, 2009). Patients with greater degrees of loneliness will focus on the progression and prognosis of their own disease and are prone to having more negative perceptions about the disease, resulting in an increased FoP. In a qualitative study including patients with breast cancer in Türkiye (Şengün İnan and Üstün, 2019) and mixed tumors in Canada (Mutsaers et al., 2016), patients with FoP stated that despite the support of their family, friends, and medical team, it was difficult for others to understand their FoP or FCR, which exacerbated the sense of loneliness. This highlights that in clinical work, attention is needed for not only the patient’s FoP but also their experience of loneliness, and the patient’s family environment and social environment should be evaluated. Medical staff can regularly organize communication between patients as patients with the same cancer experiences can better understand each other’s feelings, and staff can provide patients with a social environment that can promote their expression of emotions and ability to communicate ideas, which will help reduce patients’ loneliness and FoP.

### The relationship between hope and FoP

The level of hope in patients with gastrointestinal tumors is inversely correlated with FoP, that is, the higher the patient’s level of hope, the lower their level of FoP. Previous studies have pointed out that the hope level of cancer patients is correlated with their psychological resilience (Ye et al., 2017). In the Chinese cultural context, hope is regarded as an inner force that can energize patients in the face of adversity or difficulties, helping patients to establish positive goals and mobilize resources to actively respond to challenges and difficulties (Wu et al., 2016). A survey of breast cancer patients. Showed that patients with higher levels of hope had a lower burden of symptoms from cancer (Li et al., 2021). In addition, studies have shown that hope is an important factor in patient survival, which is related to patients’ quality of life, survival well-being, and psychological suffering (Manor-Binyamini, 2020; Shen et al., 2020). Hope includes an individual’s positive attitude toward the future, which is related to the patient’s positive attitude toward their disease. A greater level of hope can lead to the patient developing greater strength, confidence, and courage to overcome disease and can reduce the patient’s fear of the disease (Bovero et al., 2021). As a traumatic stressful event, cancer development can cause physical and psychological harm to patients that is difficult to overcome. When patients believe that disease progression is a threat, an increase in negative emotions such as anxiety and depression can weaken their confidence and hope in actively treating the disease (Kim and Kim, 2022; Yang et al., 2022). Therefore, we suggest that health care staff develop targeted interventions based on positive psychology theories to increase patients’ level of hope and reduce patients’ FoP to improve their quality of life and subjective well-being. One study showed that dignity therapy can improve the hope level of cancer patients (Zhang et al., 2022b). Clinical medical staff should evaluate and identify patients with high levels of FoP early and provide psychological counseling referral services for these patients when necessary.

## Strengths and limitations

The advantages of this research are as follows: First, by investigating the level of fear of disease in patients with gastrointestinal tumors, exploring its influencing factors, and providing a basis for research related to fear of disease progression. This can draw the attention of clinical medical staff and patients’ families to the fear of disease progression in patients with gastrointestinal tumors, and clinical caregivers can also use FOP as a screening index for patients with gastrointestinal tumors. Second, this study found that patients with high school education level, 45–59 years old, and living alone had higher levels of FOP, which is conducive to the timely detection of the high-risk group of FOP by healthcare personnel and the early adoption of intervention measures for such patients. Third, this study found a relationship between lower levels of hope, higher levels of loneliness, and high levels of fear of disease progression, which provides new ideas for subsequent research. It is suggested that other researchers may also introduce new variables to explore the relationship between them.

Nevertheless, this research has certain limitations that need to be acknowledged: First, the sample size was relatively small, which could compromise the generalizability of the research findings. Other psychological variables that might influence fear of progression have not been investigated. It should also be included that there is great variability in the time since diagnosis (as well as specific types of gastrointestinal cancer) that could affect FoP levels, leading to different periods in the treatment pathway of patients. Future studies should be based on large samples, including specific tumor types. Second, the FoP levels in patients with gastrointestinal tumors may fluctuate, and the influencing factors may also vary. Therefore, longitudinal research is needed to explore the FoP trajectory of patients with gastrointestinal tumors or to gain a deeper understanding of their true experience of fear of disease progression through qualitative research. Lastly, to ensure the quality of the questionnaire, the researchers reviewed returned questionnaires and queried patients regarding missing items; the researchers then filled in the missing items on the questionnaire. This could lead to subjectivity among researchers in some questionnaire responses.

## Conclusion

In summary, FoP is common in patients with gastrointestinal tumors. In this study, loneliness in patients with gastrointestinal tumors was found to be positively significant correlated with FoP and hope among patients with gastrointestinal tumors was inversely correlated with FoP. Education level, age, living situation, level of hope, and loneliness were independent predictors of FoP. Medical staff should assess the degree of FoP in patients with digestive tract tumors and provide more health education and social support for these patients. Targeted interventions can be formulated, such as the development of measures such as financial education and dignity therapy, to improve the hope level of patients, enhance their confidence in overcoming disease, and reduce their loneliness and FoP.

## Data availability statement

The original contributions presented in the study are included in the article/Supplementary material, further inquiries can be directed to the corresponding author.

## Ethics statement

Ethical review and approval was not required for the study on human participants in accordance with the local legislation and institutional requirements. Written informed consent from the patients was not required to participate in this study in accordance with the national legislation and the institutional requirements.

## Author contributions

YL: Conceptualization, Data curation, Supervision, Writing – original draft. TX: Methodology, Project administration, Writing – original draft. HL: Funding acquisition, Investigation, Validation, Writing – original draft. HQ: Project administration, Conceptualization, Formal analysis, Writing – review & editing. PR: Project administration, Investigation, Resources, Validation, Visualization, Writing – review & editing. XC: Conceptualization, Writing – review & editing.
