# Supplementary material for: Fear of progression, loneliness, and hope in patients with gastrointestinal cancer: a cross-sectional relational study
Source: Front Psychol. 2024 Jan 5;14:1279561. doi: 10.3389/fpsyg.2023.1279561 (PMC10796533; doi:10.3389/fpsyg.2023.1279561)
Supplement: Supplementary file 1 [file Data_Sheet_1.docx]

**Supplementary Table 1 Dummy variables setting**

| **Dumb variables** | **Assignment mode** |
| --- | --- |
| Scores of FOP-Q-SF | Enter with original value |
| Scores of CLS | Enter with original value |
| Scores of HHI | Enter with original value |
| Educational level | Primary or below（Z_1_=0，Z_2_=0,Z_3_=0,Z_4_=0）；Junior high school（Z_1_=0，Z_2_=1,Z_3_=0,Z_4_=0）；High school（Z_1_=0，Z_2_=0,Z_3_=1,Z_4_=0）；College or above（Z_1_=0，Z_2_=0,Z_3_=0,Z_4_=1） |
| Age group | ≥60（Z_1_=0，Z_2_=0,Z_3_=0）；45~59（Z_1_=0，Z_2_=1,Z_3_=0）；<45（Z_1_=0，Z_2_=0,Z_3_=1） |
| Marital status | Married（Z_1_=0，Z_2_=0,Z_3_=0）；Widow（Z_1_=0，Z_2_=1,Z_3_=0）；Divorce（Z_1_=0，Z_2_=0,Z_3_=1） |
| Scores of NRS | No pain（Z_1_=0，Z_2_=0,Z_3_=0,Z_4_=0）；Mild pain（Z_1_=0，Z_2_=1,Z_3_=0,Z_4_=0）；Moderate pain（Z_1_=0，Z_2_=0,Z_3_=1,Z_4_=0）；Severe pain（Z_1_=0，Z_2_=0,Z_3_=0,Z_4_=1） |
| Working conditions | Unemployment（Z_1_=0，Z_2_=0,Z_3_=0,Z_4_=0）；On the job（Z_1_=0，Z_2_=1,Z_3_=0,Z_4_=0）；Farmer（Z_1_=0，Z_2_=0,Z_3_=1,Z_4_=0）；Retirement（Z_1_=0，Z_2_=0,Z_3_=0,Z_4_=1） |
| Monthly income (yuan) | <1000（Z_1_=0，Z_2_=0,Z_3_=0）；1000~2999（Z_1_=0，Z_2_=1,Z_3_=0）；≥3000（Z_1_=0，Z_2_=0,Z_3_=1） |
| Geographic area | Rural=0；Urban=1 |
| Living status | Live with family =0；Live alone =1 |
| Type of Insurance | Social insurance =0；Rural cooperative medical insurance =1 |

Abbreviations: FoP-Q-SF, Fear of Progression Questionnaire⁃Short Form ; HHI , Herth Hope Index ; CLS , Cancer Loneliness Scale ; NRS, Numerical Rating Scale

**Supplementary Table 2. Mean scores and standard deviations of the FoP-Q-12 items.**

| Item | Mean | SD |
| --- | --- | --- |
| 1. Being afraid of disease progression | 3.17 | 1.088 |
| 2. Being nervous prior to doctors’ appointments or periodic examinations | 2.29 | 1.321 |
| 3. Being afraid of pain | 2.85 | 1.307 |
| 4. Being afraid of becoming less productive at work | 2.60 | 1.418 |
| 5. Having physical symptoms, e.g., rapid heartbeat, stomach ache, nervousness | 2.53 | 1.360 |
| 6. Being afraid by the possibility that the children could contract cancer | 2.42 | 1.348 |
| 7. Being afraid of relying on strangers for activities of daily living | 1.61 | 1.284 |
| 8. Being afraid of no longer be able to pursue hobbies | 2.75 | 1.274 |
| 9. Being afraid of severe medical treatments in course of the illness | 3.33 | 0.958 |
| 10. Worrying that medications could damage the body | 3.65 | 1.003 |
| 11. Worrying about what will become of the family if something should happen to the patient | 3.40 | 1.298 |
| 12. Being afraid of not being able to work anymore | 2.34 | 1.475 |

SD: standard deviation.
